# A systematic literature review on the use of multicriteria decision making methods for small and medium-sized enterprises innovation assessment

**DOI:** 10.3389/frai.2025.1605756

**Published:** 2025-07-11

**Authors:** Mayra Leticia Rodríguez-Carrillo, Luis Pérez-Domínguez, Roberto Romero-López, David Luviano-Cruz, Ernesto León-Castro

**Affiliations:** ^1^Department of Industrial Engineering and Manufacturing, Institute of Engineering and Technology, Universidad Autónoma de Ciudad Juárez, Ciudad Juárez, Chihuahua, Mexico; ^2^Faculty of Economics and Administrative Sciences, Universidad Católica de la Santísima Concepción, Concepción, Chile

**Keywords:** systematic literature review, multicriteria decision making methods, small and medium-sized enterprises, innovation, PRISMA method

## Abstract

Multi-criteria decision making (MCDM) methods are essential tools for assessing multiple factors in various contexts, including innovation in small and medium-sized enterprises (SMEs). In this study, a systematic literature review (SLR) was conducted based on a literature search in Web of Science, Scopus and Google Scholar, covering the period 2018–2024, taking as a basis the general guidelines and main phases of an SLR, in addition, the Preferred Reporting Items for Systematic reviews and Meta-Analyses (PRISMA) method was used, which allowed the selection of 25 relevant articles. From the analysis, four main trends in innovation assessment were identified: Innovation Capacity and Business Strategies, Open Innovation, Evaluation and Management, Technological and Digital Innovation, and Green Innovation and Sustainability. The results indicate that India and China are the countries with the highest volume of publications on this topic, while the business and academic sectors are the most studied, followed by the social sector. In addition, other key factors assessed in SMEs using MCDM methods were identified, grouped into five main themes including industry 4.0 and digital transformation, sustainability and green manufacturing, risk management and business resilience, decision making in trade and markets, and business management strategies and technology selection, broken down into 11 specific approaches. The review shows that assessing innovation in SMEs requires a multidisciplinary and collaborative approach tailored to business needs. It also shows a preference for fuzzy tools and the combination of different MCDM methods. This article provides an updated diagnosis on the use of multiple criteria in the innovation assessment in SMEs, providing a basis for future research and applications in this field.

## 1 Introduction

Small and medium-sized enterprises (SMEs) are crucial to the economic development of countries, serving as one of the main sources of employment (Gay and Szostak, 2019). Their organizational structure allows for faster decision-making and facilitates adaptation to environmental changes (Ibidunni et al., 2020). The essential role of SMEs in global economies is widely acknowledged (Algan, 2019), and as a result, governments worldwide have increasingly focused on promoting and supporting their growth as part of national development strategies. SMEs not only stand out due to their large number but also because of their impact as primary engines of employment, economic growth, and innovation. According to the World Economic Forum (2023), SMEs represent over 90% of all businesses worldwide, generate between 60 and 70% of total employment, and contribute ~55% of the Gross Domestic Product (GDP) in developed economies.

Despite their importance, SMEs often operate at a disadvantage compared to large corporations, especially in terms of access to financing (Bui et al., 2021), which limits their competitiveness, development and capacity for innovation (López and Antelo, 2010). To better understand the challenges they face, it is essential to consider the regional differences that condition their performance. In Latin America, for example, SMEs are affected by high levels of informality and unstable regulatory frameworks (Barbero and Vidal Olivares, 2022); in Asia, they face the challenge of integrating into global value chains in contexts of rapid digitalization (Vu et al., 2022); in Africa, they are grappling with limitations in infrastructure and access to financial services (Muriithi, 2017); while in Europe and North America, the challenges are focused on sustainability, digital transformation and global competition (Omrani et al., 2022; Ragazou et al., 2022).

This diversity of scenarios highlights the need for differentiated and contextualized approaches when analyzing the role and evolution of SMEs globally, especially with regard to their ability to innovate and adapt to changing environments. Innovation is a particularly critical factor for the survival, development, and adaptability of SMEs in an increasingly competitive, dynamic, and uncertain environment (Toledo et al., 2022). To remain competitive, SMEs must adopt innovative strategies that transform their processes, organizational structures and business models (Cosenz and Bivona, 2021). Innovation in this context refers to the effective use of internal and external resources to develop new products, services, processes, or systems in response to changes in markets, technologies, and competition (Rumanti et al., 2022; Saunila, 2014).

However, innovation is not a simple task. It involves complex knowledge-related activities, such as creation, diffusion and application, which add value to the organization (Chien et al., 2021a; Enjolras et al., 2020; Grillo et al., 2018). SMEs face several obstacles when pursuing innovation, such as limited financial, technological and human resources, and the lack of effective tools to support decision making in complex contexts (Islam et al., 2021). In this regard, MCDM methods have gained attention as useful tools to address such complexity. These methods are a very accurate tool to make decisions considering different qualitative and quantitative criteria (Dhurkari, 2022) and for SMEs they allow evaluating innovation from multiple perspectives -economic, financial, managerial and strategic- supporting the development of more solid decisions (Taherdoost and Madanchian, 2023).

In today's data-driven business environment, all organizations, including SMEs, must make strategic decisions based on relevant and often conflicting information (Xu et al., 2017). This decision-making process is inherently complex due to the interaction between multiple, sometimes contradictory, elements. Consequently, various analytical tools have been developed and adopted in organizational and industrial contexts to evaluate and compare alternatives based on multiple criteria (Peng et al., 2017; Gonçalves et al., 2019). For SMEs in particular, MCDM methods offer a systematic framework for informed and strategic decision-making in areas such as resource allocation, innovation, and business growth (Bhatia and Diaz-Elsayed, 2023). These methods help evaluate alternatives using diverse criteria, such as cost, quality, and sustainability (Roy and Shaw, 2023), and provide a structured approach that integrates both quantitative and qualitative dimensions of decision-making (Singh and Pant, 2021; Wątróbski et al., 2019).

Moreover, the versatility of MCDM methods allows their application in diverse organizational scenarios, including supplier selection, project evaluation, technology adoption, and overall strategic planning (Korcsmáros and Csinger, 2022). Within the context of SMEs, MCDM approaches help classify, assess, and compare alternatives toward a common goal, where each alternative is assessed based on a set of weighted criteria (Gupta and Barua, 2018a). This structured assessment helps SMEs overcome the challenges of decision making and improves their capacity for innovation.

Although MCDM methods have gained increasing relevance in evaluating organizational performance, their specific application in SMEs particularly in the context of innovation remains fragmented and underexplored. While some studies have successfully applied MCDM methods to assess technological adoption or competitiveness in SMEs (Chang et al., 2021; Hung et al., 2011), the academic literature still lacks a recent, systematic, and comprehensive review that consolidates the main trends and findings in this field (Saunila, 2020). The integration of MCDM in innovation assessment is often addressed in isolation or combined with broader frameworks such as TOE; yet these approaches rarely offer a consolidated mapping of how such methods contribute to strategic innovation management within SMEs (Gupta and Barua, 2018b; Chang et al., 2021). Consequently, there is a pressing need for a more structured synthesis that explores both the methodological contributions of MCDM methods and their practical implications across various strategic dimensions relevant to SMEs.

Given this gap, the present study aims to provide a systematic literature review (SLR) to address two core research questions:

- Q1. What are the main trends and findings in the application of MCDM methods for innovation assessment in SMEs?
- Q2. What other factors, beyond innovation, have been evaluated in SMEs using MCDM methods?

The main contribution of this review lies in the identification of four key trends in the use of MCDM methods in SME innovation contexts: (1) innovation capacity and business strategies, (2) open innovation, (3) technological and digital innovation, and (4) green innovation and sustainability. In addition, the study reveals an increasing use of these methods in broader organizational areas, highlighting new opportunities for both future academic research and practical applications.

This article is structured as follows: first, the methodology for conducting SLR is presented, following the main phases proposed by Kitchenham et al. (2010) of systematic review protocols. Subsequently, the research questions and the methodology used to answer them are defined. Next, the search strategy is described, as well as the inclusion and exclusion criteria, based on the Preferred Reporting Items for Systematic reviews and Meta-Analyses (PRISMA) method (Haddaway et al., 2022). Subsequently, a descriptive analysis of the selected publications is performed, covering aspects such as the year of publication of the selected articles, the country of origin, the authors with the highest number of publications, the most frequently used MCDM methods, and the thematic trends. Based on these trends, the selected studies are classified into four main analytical sections. Finally, the article concludes with a discussion of the main contributions and implications, and suggestions for future research.

## 2 Methods

**Figure 1:**
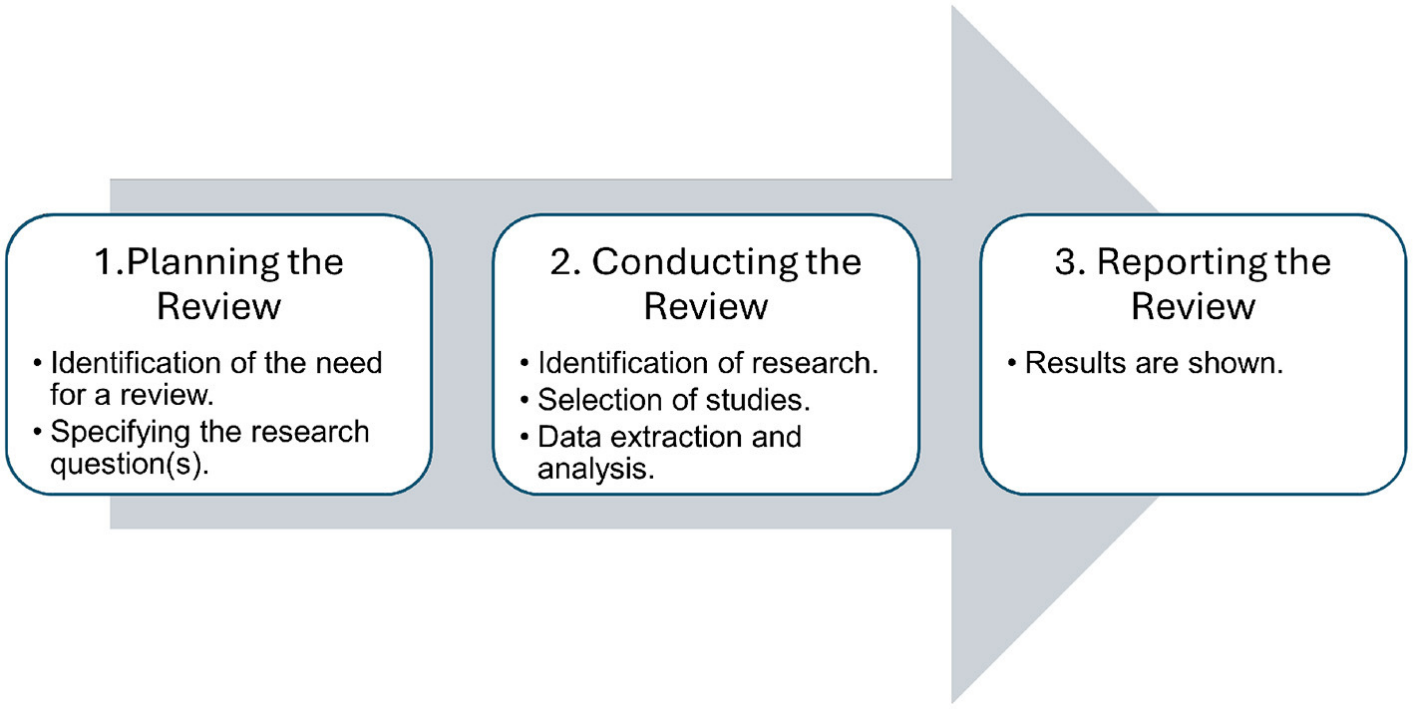
Stages in a systematic review.

This study employs an SLR, which is a method used to identify, analyze and interpret all available research relevant to a specific research question, topic area or phenomenon of interest (Kitchenham et al., 2010). Essentially, this type of review involves an exhaustive search for significant contributions on a particular topic, which are then evaluated and synthesized following a clear and pre-established methodology. An SLR is particularly suitable for this study because it ensures transparency, replicability and comprehensiveness in the collection and analysis of existing literature, thereby minimizing bias and increasing the reliability of the findings. For its development, a series of well-defined stages are rigorously followed, involving three main phases: Planning the Review, Conducting the Review using the PRISMA method (Page et al., 2021), and Reporting the Review, stages of systematic review, as shown in Figure 1, as proposed by Kitchenham et al. (2010).

### 2.1 Planning the review

The initial stage of the systematic review corresponds to the planning phase. Following the process established by Kitchenham et al. (2010), it was identified that there are few literature reviews focused on the topic of interest, specifically on the use of MCDM methods for the innovation assessment in the context of SMEs. Consequently, two research questions were formulated to explore the state of the art on this topic, and a proposed solution was established.

#### 2.1.1 Identification of the need for a review

In recent years, several studies have been published applying MCDM methods to evaluate different strategic aspects in organizational environments, such as supplier selection (Sahoo et al., 2024), sustainability analysis (Chowdhury and Paul, 2020), project prioritization (de Souza et al., 2021), and risk management (de Almeida et al., 2017). Some previous reviews have systematized these applications in sectors such as manufacturing (Mazumdar et al., 2023), energy (Siksnelyte-Butkiene et al., 2020) or environmental management (Sahoo et al., 2024). However, upon conducting an exploratory literature review, it was not possible to identify systematic reviews that explicitly link MCDM methods to innovation assessment in the specific context of SMEs.

This gap is particularly relevant because SMEs face specific challenges, such as the limited availability of resources and capabilities to implement complex analytical tools, even though they must make critical innovation decisions to stay competitive in dynamic environments. In this context, the application of MCDM methods represents a valuable opportunity to facilitate strategic decisions based on multiple criteria; however, its use has not yet been comprehensively characterized in the field of innovation within this sector.

Therefore, this review is proposed as an original and necessary contribution to integrate, analyze and make visible the use of MCDM methods in the study of innovation in SMEs, providing a frame of reference to understand the current state of knowledge, identify emerging patterns and guide future lines of research.

#### 2.1.2 Research questions

The present study aims to offer a SLR to answer two key research questions:

Q1. What are the main trends and findings in the application of MCDM methods for innovation assessment in SMEs?

To answer this question, articles were searched in specialized databases such as Scopus, Web of Science, and Google Scholar. Keywords such as MCDM methods, innovation, SMEs, innovation capability, decision-making techniques, among others, were used. The analysis of the identified articles will allow for the establishment of key trends in the use of MCDM methods to innovation assessment in SMEs. The aspects to be examined include: MCDM methods used, innovation assessment criteria, recent trends, and key findings in the application of MCDM methods for innovation. The objective is to provide a detailed and structured overview of how MCDM methods has been used to assess innovation in SMEs, identifying patterns, gaps in the literature, and potential future research directions.

Q2. What other factors, besides innovation, have been evaluated in SMEs using MCDM methods?

To answer this question, a literature analysis similar to the one conducted for the first research question will be carried out, but with a broader focus, covering studies that apply MCDM methods to evaluate various aspects of SMEs. Based on the publications identified in the previous analysis, other factors assessed using these methods will be identified, such as financial and economic performance, sustainability and corporate social responsibility, risk management, digital transformation and technology adoption, etc. This review is expected to provide a comprehensive understanding of the applicability of MCDM methods in strategic decision-making for SMEs, helping to determine which factors have been prioritized and which could be explored in future research.

### 2.2 Conducting the review

#### 2.2.1 Terms and search process

The exploratory search on the topic of the research is initiated using the computer program Publish or Perish, which allows the analysis of data sources and academic citations referring to the topic of interest, for this it was selected in the options of the program that the search was in the databases of Scopus, Google Scholar and Web of Science, incorporating the following terms in the title, abstract or keyword of the search equation: “Multicriteria Decision Making”; “MCDM”; “Multi-Criteria Decision Analysis”; “Small and Medium-Sized Enterprises”; “SMEs”; “Innovation”; “Innovative Capacity”; “Innovat.” This exploration yielded a total of 8,543 results; however, to validate the search equation, a pilot test was carried out to check if making changes to the equation would yield new results. Table 1 shows the database-specific search strings and the number of papers found.

**Table 1:** Database search results for information.

**Search keywords**	**Database**	**Amount**
TITLE-ABS-KEY((“Multicriteria decision making” OR “MCDM” OR “Multi-criteria decision analysis”) AND (“Small and medium-sized enterprises” OR “Small and medium enterprises” OR “SMEs”) AND (“Innovation” OR “Innovative capacity” OR “Innovat*”))	SCOPUS	105
(“Multicriteria decision making” OR MCDM OR “Multi-criteria decision analysis”) AND (“Small and medium enterprises” OR “Small and medium-sized enterprises” OR SMEs) AND (“Innovation” OR “Innovative capacity” OR Innovat*)	GOOGLE SCHOOLAR	8,394
TS=(“Multicriteria decision making” OR “MCDM” OR “Multi-criteria decision analysis”) AND TS=(“Small and medium enterprises” OR “Small and medium-sized enterprises” OR “SMEs”) AND TS=(“Innovation” OR “Innovative capacity” OR Innovat*)	WOS	44

**Figure 2:**
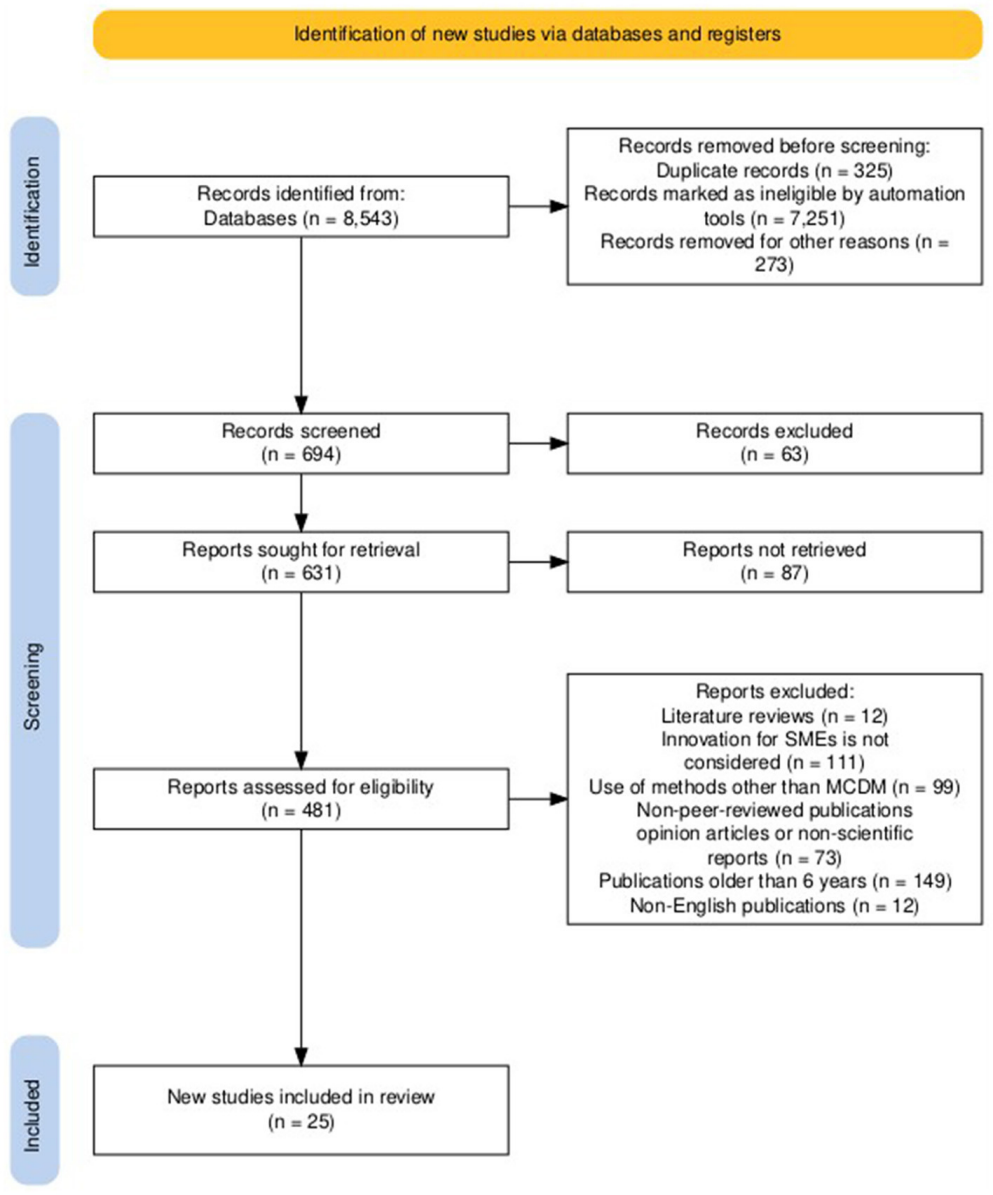
Selection of references based on the PRISMA method.

On the other hand, Figure 2 shows the PRISMA flow chart (Page et al., 2021) that describes the methodological rigor applied during the selection process of the studies included in this systematic review. An initial group of 8,543 records was identified through comprehensive database searches. After the elimination of duplicate records (*n* = 325), records excluded by automated tools (*n* = 7,251) and others deemed ineligible for various reasons (*n* = 273), a total of 694 records went to the selection phase. After the selection of titles and abstracts, 63 records were excluded and 631 full-text articles were requested for retrieval. Of these, 150 could not be accessed. The remaining 481 articles underwent a detailed eligibility assessment, which resulted in the exclusion of 456 articles due to reasons including: lack of relevance to innovation in SMEs (*n* = 111), use of methods other than the MCDM (*n* = 99), non-peer-reviewed or non-scientific nature (*n* = 73), date of publication obsolete (more than six years; *n* = 149), non-English language (*n* = 12), and being bibliographic reviews (*n* = 12). Consequently, 25 studies met all inclusion criteria and were incorporated into the final analysis.

#### 2.2.2 Item selection method

The following inclusion and exclusion criteria were considered in the articles found:

Inclusion criteria:

- The publication dates of the selected works are between 2018 and 2024.
- Only works written in English are included.
- The type of publication to be considered includes scientific articles or conference articles.
- The articles selected from the search obtained should clearly focus on the application of MCDM methods on solution strategies for innovation in SMEs.

Exclusion criteria:

- Literature reviews are not included, but they are taken as a reference to establish comparisons with the findings of the review.
- References to the use of the MCDM methods in industries other than SMEs are excluded.
- Articles that do not use MCDM methods in SMEs or that do not specify the MCDM method used are excluded.
- Sources that cannot be accessed in the publication or at least in the abstract of the research are excluded.
- Non-English publications are excluded.

#### 2.2.3 Select papers

**Figure 3:**
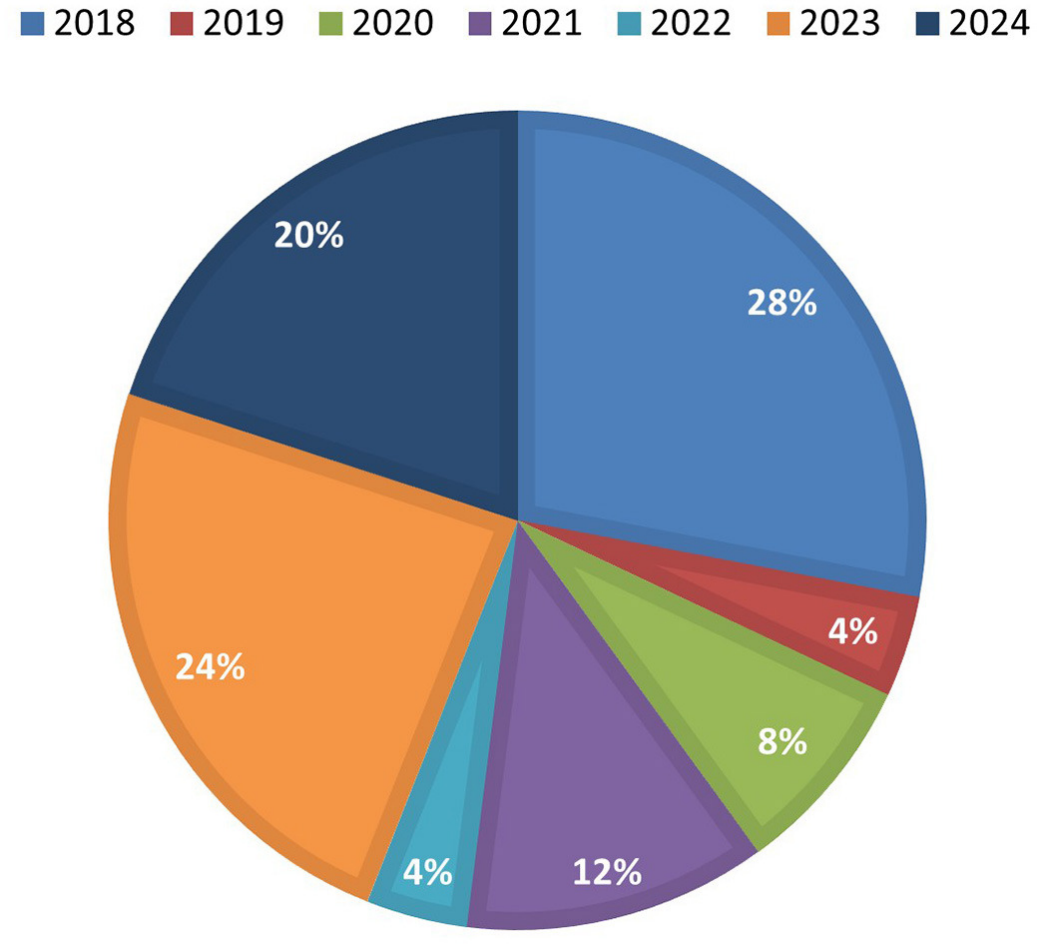
Select papers per year.

Table 2, shows the 25 articles selected for the analysis and data extraction, while in Figure 3, it is possible to appreciate the distribution by year of these documents, it can be seen that most of the articles analyzed were published in 2018, however, from this year onwards it shows an upward trend in subsequent years, indicating a growing interest in the research of this topic.

**Table 2:** Papers included in the SLR.

**No**.	**References**	**MCDM methods**	**Title**
**1**	**Matroushi et al., 2018**	**AHP**	**Prioritizing the factors promoting innovation in Emirati female-owned SMEs: AHP approach**
2	Rahmanita et al., 2018	FANP/TOPSIS	Adaptive FANP and TOPSIS method for innovation strategy of SME
3	Kiron and Kannan, 2018	FANP	Application of fuzzy analytical network process for the selection of best technological innovation strategy in steel manufacturing SMEs
4	Grillo et al., 2018	Cognitive mapping/DEX	A knowledge-based innovation assessment system for SMEs: adding value with cognitive mapping and MCDA
5	Macedo Filho and Almeida, 2018	AHP/TOPSIS	Measuring and evaluating innovation management in SMEs: proposition of a multicriteria model for selecting indicators and metrics
6	Gupta and Barua, 2018b	BWM/FTOPSIS	A novel hybrid multi-criteria method for supplier selection among SMEs on the basis of innovation ability
7	Gupta and Barua, 2018a	BWM/FTOPSIS	A framework to overcome barriers to green innovation in SMEs using BWM and Fuzzy TOPSIS
8	Singh et al., 2019	AHP	Justification of technology innovation implementation in Indian SMEs using AHP
9	Enjolras et al., 2020	AHP/VIKOR	Evaluating innovation and export capabilities of SMEs: toward a multi-criteria decision-making methodology
10	Musaad O et al., 2020a	FAHP/FTOPSIS	A fuzzy multi-criteria analysis of barriers and policy strategies for SMEs to adopt green innovation
11	Chou et al., 2023	FDELPHI/ANP	Innovation strategy development and facilitation of an integrative process with an MCDM framework
12	Chien et al., 2021a	AHP/TOPSIS	Assessing the prioritization of barriers toward green innovation: SMEs Nexus
13	Hakaki et al., 2021	FDEMATEL/ANT COLONY	An optimized model for open innovation success in manufacturing SMES
14	Chen et al., 2022	DEMATEL/DANP	Integrating the MCDM method to explore the business model innovation in Taiwan: A case study in affiliated restaurants
15	Amoozad Mahdiraji et al., 2023	FDELPHI/DEMATEL/ANP/SWARA	Toward financing the entrepreneurial SMEs: exploring the innovation drivers of successful crowdfunding via a multi-layer decision-making approach
16	Yıldırım et al., 2023	FISM/MICMAC/DEMATEL	Why can SMEs not adopt green innovation? An assessment via fuzzy ISM-MICMAC-DEMATEL
17	Vakil Alroaia, 2023	ANP/PROMETHEE	Open innovation and SMEs: providing a model for business development (an application on Iranian industrial park)
18	Singh, 2023	MATLAB fuzzy logic toolbox	Evaluation of technological innovation initiatives for Indian SMEs using the fuzzy-based model
19	Farjam et al., 2023	BWM/FTODIM	A conceptual model for open innovation risk management based on the capabilities of SMEs: a multi-level fuzzy MADM approach
20	Albahri et al., 2023	q-RPFWZIC/SAW	Evaluation of organizational culture in companies for fostering a digital innovation using q-rung picture fuzzy based decision-making model
21	Shahin et al., 2024	IFMBWM/IVIF-MULTIMOORA	Identifying and prioritizing the barriers to green innovation in SMEs and the strategies to counteract the barriers: An interval-valued intuitionistic fuzzy approach
22	Mutlag et al., 2024	AHP	Integration SMEs' growth characteristics vs. innovation alternative solutions using multi-criteria
23	Jing et al., 2024	DEMATEL/DANP/VIKOR	Advancing an evaluation model: how do family SMEs select innovation scheme in lean management?
24	Torbacki, 2024	DEMATEL/PROMETHEE II	A framework for assessing innovations, business models and sustainability for Software companies using hybrid MCDM
25	Moreira et al., 2024	AHP	Potential for frugal innovation in a Brazilian regional system: a study based on a multicriteria approach

## 3 Reporting the review—Results

### 3.1 Select papers per year

The Figure 3 shows the percentage distribution by year of the articles selected for the analysis of the use of MCDM methods for SMEs Innovation Assessment, classified by year of publication. The year with the highest number of publications was 2018, representing 28% of the total with seven articles. It was followed by 2023 with 24% (six articles) and 2024 with 20% (fiv articles). In 2021, three articles were registered (12%), while in 2020, 2 were selected (8%). Finally, 2019 and 2022 presented the lowest number of studies included, with only one article each (4%). This distribution reflects a relevant concentration of recent research and a growing interest in recent years in applying MCDM methods to analyze innovation in SMEs.

### 3.2 Papers by country

**Figure 4:**
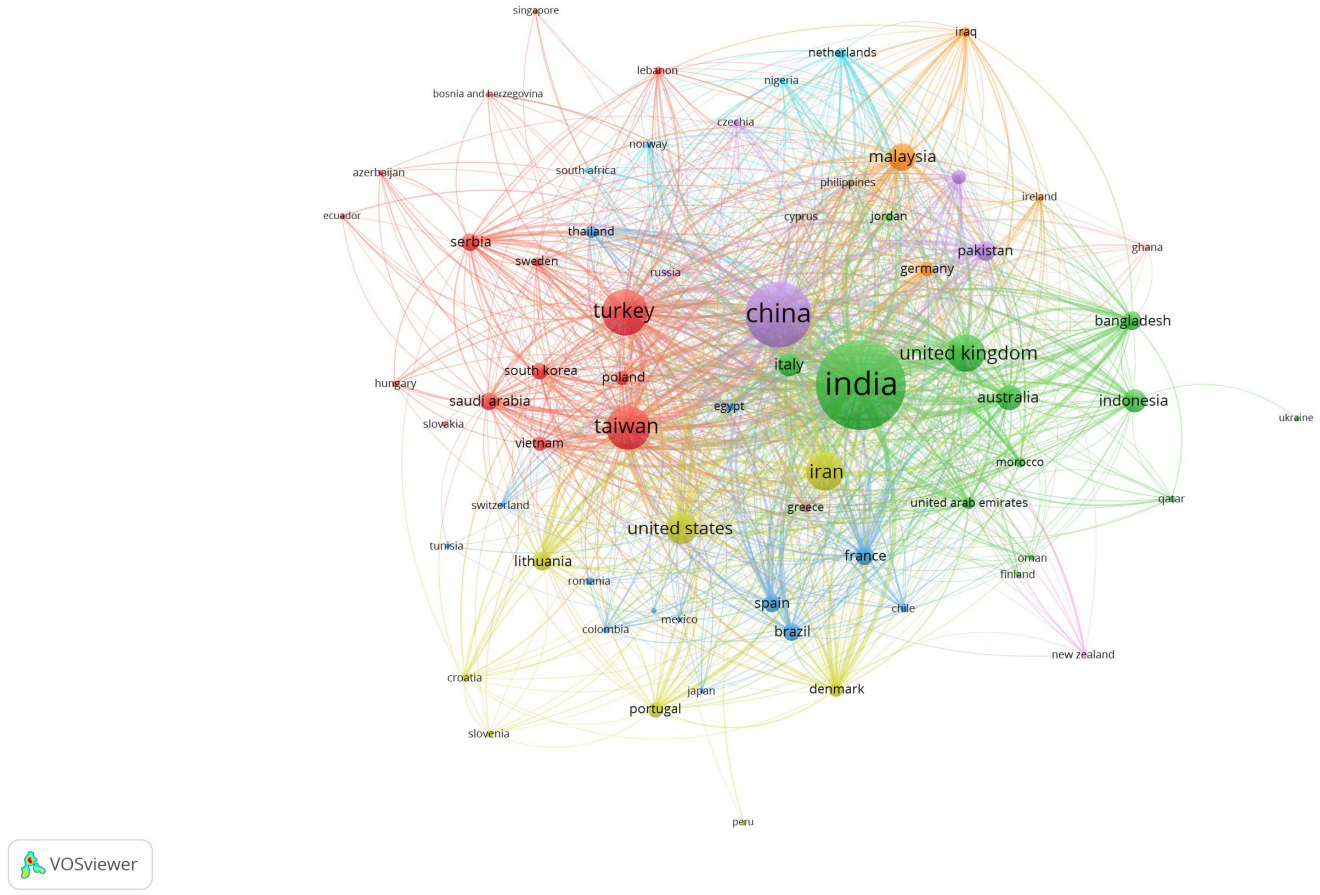
Countries with the highest presence in research.

The selected articles were published in various countries, and are distributed as follows: India is the country with the most publications (five articles), China (four articles), Iran (four articles), Brazil (two articles), also with only one publication, the countries of Indonesia, France, Iraq, Malaysia, Poland, Taiwan, Turkey, United Kingdom, United Arab Emirates, and United States are included. Figure 4 presents a visual representation of the countries with the highest presence in research related to the topic of interest. The size of the circles indicates the number of publications, so that larger circles correspond to a greater number of publications compared to those of smaller size.

### 3.3 Most relevant sources

**Figure 5:**
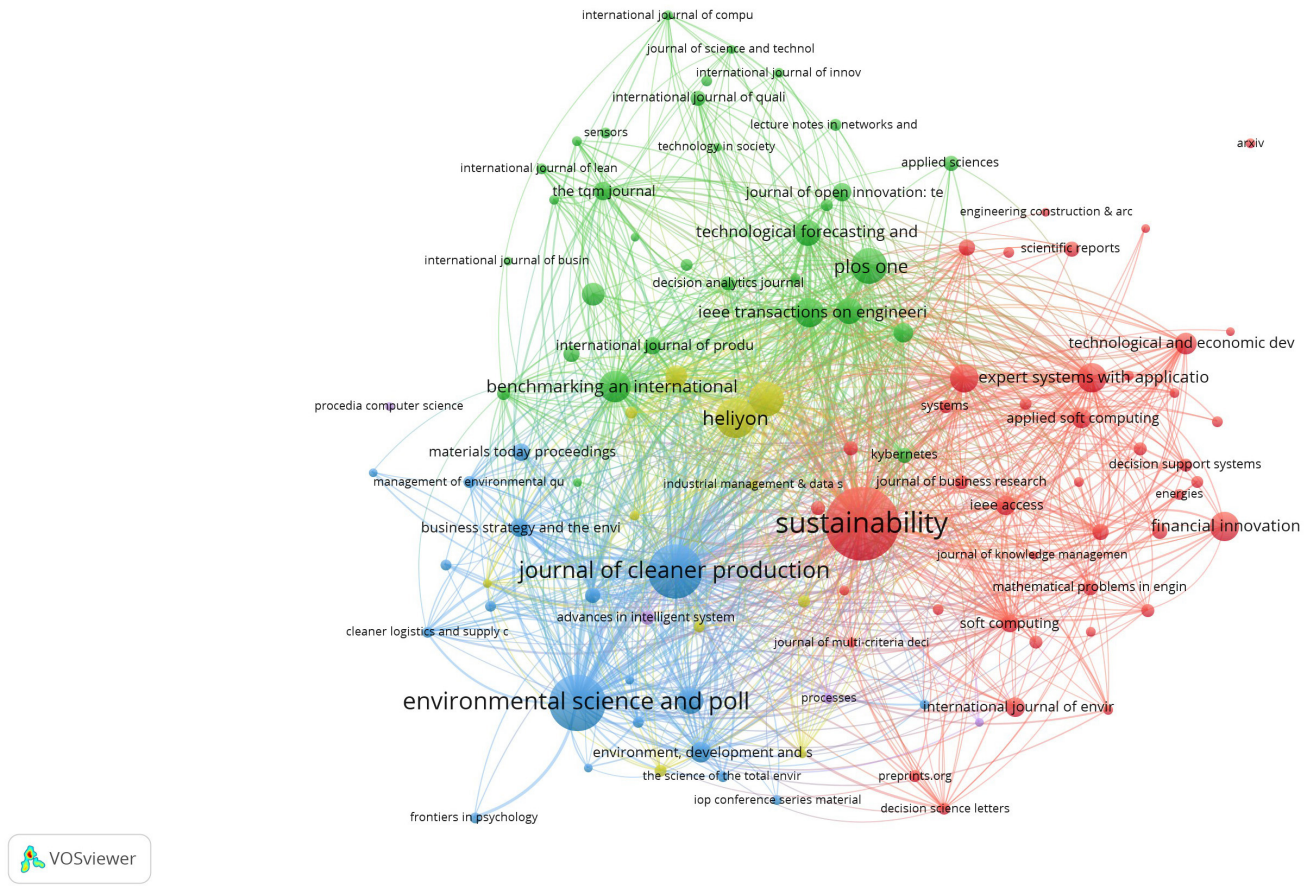
Most relevant sources.

The journals with the highest number of publications on the use of MCDM methods for the assessment of innovation in SMEs belong mainly to areas related to sustainability, environmental sciences and pollution, clean production and environmental care in general. Secondly, there are those with a focus on technology, while, to a lesser extent, the publications come from journals specialized in business and administrative strategies. In the Figure 5, the size of the circles represents the number of publications in each thematic category, so that a larger size indicates a greater number of studies found in that area.

### 3.4 Authors with the highest number of publications

**Figure 6:**
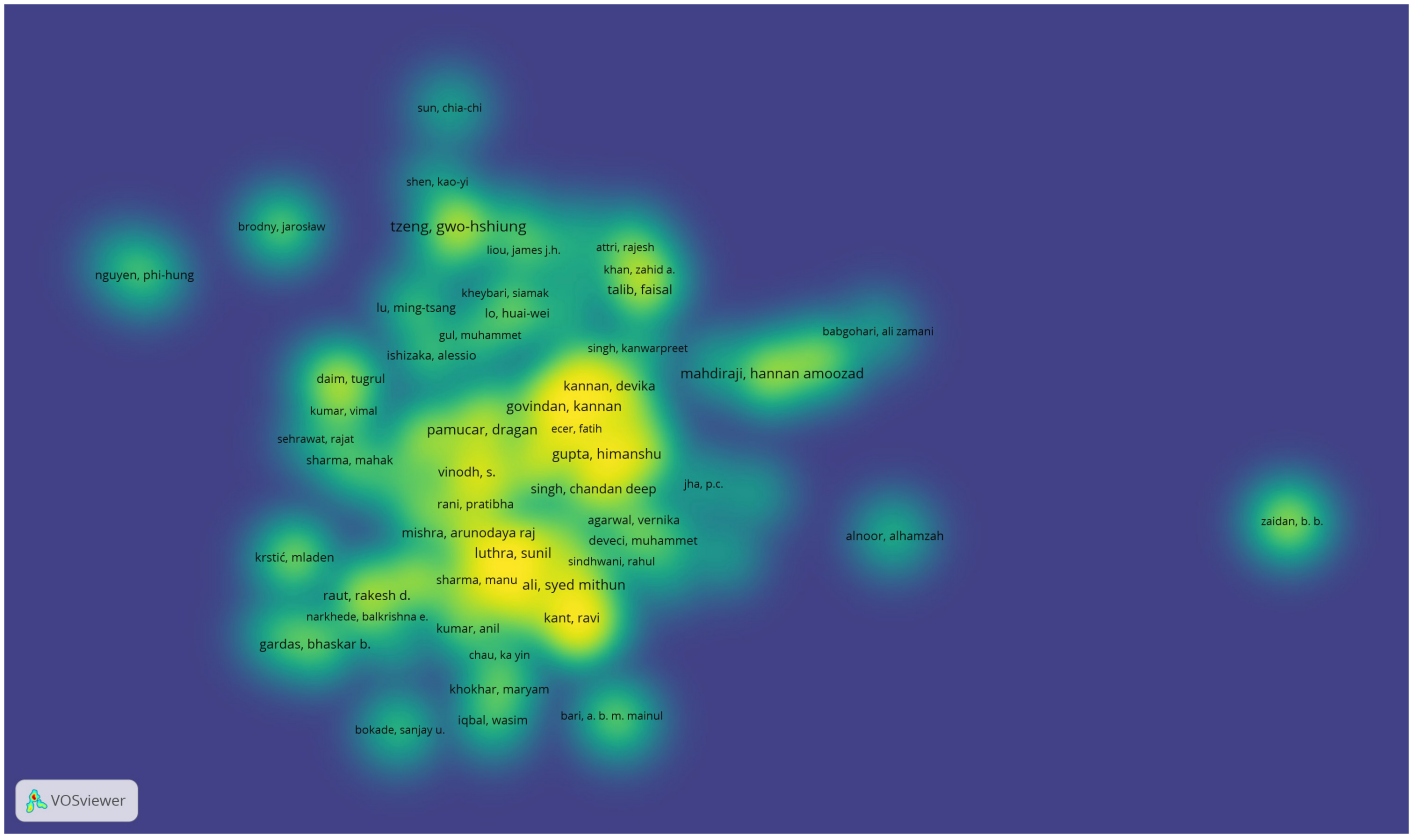
Most relevant authors.

Numerous authors have contributed research on the use of MCDM methods in the context of SMEs, particularly in issues related to innovation. As in the country analysis, it is observed that the largest presence of authors comes from India and China, which reflects the interest and development of these approaches in these regions. In Figure 6, this pattern is visualized through the heat map, where the density of the presence of the authors is represented graphically, the most marked areas on the map allow to identify more clearly those authors who concentrate most of the academic production in this field.

### 3.5 Methodologies used in selected papers

**Figure 7:**
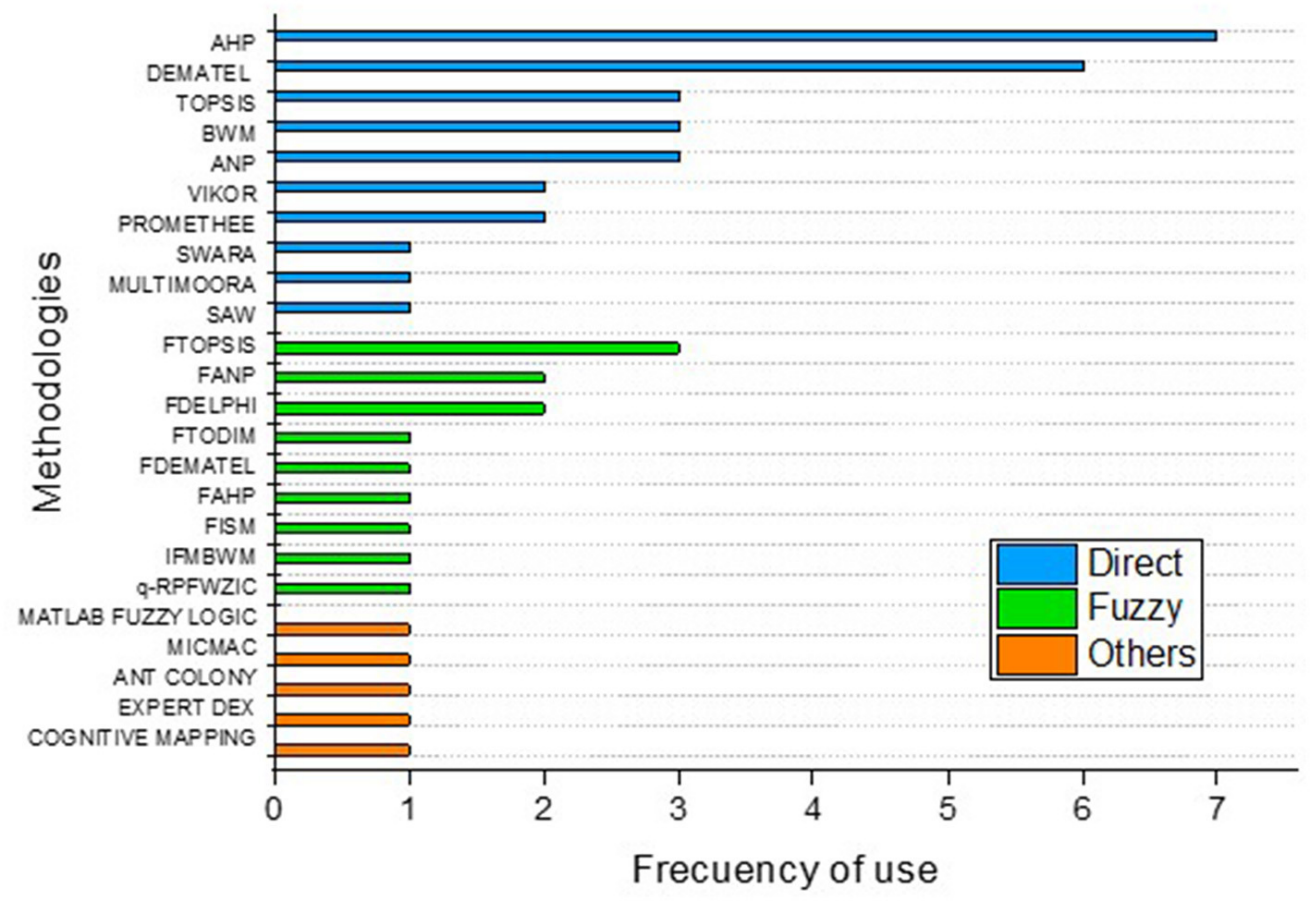
Methodologies used in selected papers.

There are several methods for assessing innovation in SMEs (Figure 7), in the review of selected academic works, it was observed that only five used a single multi-criteria methodology, compared to the remaining twenty where a combination of several methods was used, the most used direct method is the Analytic Hierarchy Process (AHP) with seven mentions, then by Decision-Making Trial and Evaluation Laboratory (DEMATEL) with 6, followed by Technique for Order Preference by Similarity to Ideal Solution (TOPSIS), Best-Worst Method (BWM) and Analytic Network Process (ANP) with 3, then Vise Kriterijumska Optimizacija I Kompromisno Resenje (VIKOR) and Preference Ranking Organization Method for Enrichment Evaluation (PROMETHEE) with 2, and finally Step-Wise Weight Assessment Ratio Analysis (SWARA), Multi-Objective Multi-Attribute Optimization and Ratio Analysis (MULTIMOORA), and Simple Additive Weighting (SAW) with 1, it should also be noted that at least half of the works are based on the use of fuzzy tools as a fundamental part of their analysis, the most commonly used fuzzy tool is Fuzzy Technique for Order Preference by Similarity to Ideal Solution (FTOPSIS) found in three works, followed by Fuzzy Analytic Network Process (FANP) and Fuzzy Delphi Method (FDELPHI) with two and Fuzzy TODIM (FTODIM—an acronym in Portuguese for Interactive and Multicriteria Decision Making–), Fuzzy Decision-Making Trial and Evaluation Laboratory (FDEMATEL), Fuzzy Analytic Hierarchy Process (FAHP), Fuzzy Interpretive Structural Modeling (FISM), in addition, other combinations of fuzzy methods such as Intuitionistic Fuzzy Multiplicative Best-Worst Method (IFMBWM), and q-rung picture fuzzy-weighted zero-inconsistent (q-RPFWZIC) with a mention. Fuzzy logic-based techniques provide an effective way to address complex problems with inherent uncertainty, making them the preferred choice for many authors (Wang et al., 2016).

Other methods used that have a mention respectively are Impact Matrix Cross-Reference Multiplication Applied to a Classification (MICMAC), optimization by ant colony, Cognitive Mapping, which is a technique to represent and analyze thought structures and use of software such as Decision Expert as a support for decision making, and MATLAB Fuzzy Logic Toolbox, which is the use of the fuzzy logic toolbox. Most authors use more than one MCDM method to carry out their analyses. This combination of approaches allows researchers to leverage the strengths of different techniques, which in turn contributes to more accurate and comprehensive results. The trend toward the use of multiple MCDM methods reflects an approach focused on comprehensive and adaptive solutions to complex decision-making problems (Singh and Pant, 2021). By combining various methodologies, authors can approach challenges from various perspectives, thereby improving the quality and reliability of their conclusions.

### 3.6 Main trends and findings in the application of MCDM methods for the assessment of innovation in SMEs

Table 3 summarizes the main trends, methods, and findings identified in studies that apply MCDM methods to assess innovation in SMEs, grouped into four key approaches.

**Table 3:** Main trends and findings in the application of MCDM methods for the assessment of innovation in SMEs.

**Trend/focus area**	**Key MCDM methods and references**	**Key findings**
Innovation capacity and business strategies	AHP (Matroushi et al., 2018; Moreira et al., 2024; Mutlag et al., 2024), FANP (Rahmanita et al., 2018), TOPSIS (Kiron and Kannan, 2018), DEX (Grillo et al., 2018), BWM + FTOPSIS (Gupta and Barua, 2018b), FDELPHI + ANP + DEMATEL + DANP (Chou et al., 2023; Chen et al., 2022)	Strategic flexibility, access to finance, human capital, and supportive policies are fundamental for fostering innovation in SMEs
Open innovation, evaluation and management	AHP + TOPSIS (Macedo Filho and Almeida, 2018), FDEMATEL + Ant Colony (Hakaki et al., 2021), FDELPHI + DEMATEL + ANP + SWARA (Amoozad Mahdiraji et al., 2023), ANP + PROMETHEE (Vakil Alroaia, 2023), BWM + FTODIM (Farjam et al., 2023), DEMATEL + DANP + VIKOR (Jing et al., 2024)	Highlights the importance of systematic evaluation, strategic management, risk mitigation, and institutional collaboration in open innovation
Technological and digital innovation	AHP (Singh et al., 2019; Enjolras et al., 2020), VIKOR (Enjolras et al., 2020), Fuzzy phase model (Singh, 2023), Fuzzy image-based model (Albahri et al., 2023)	Technological innovation improves performance and competitiveness; digital adoption driven by entrepreneurial culture
Green innovation and sustainability	BWM + FTOPSIS (Gupta and Barua, 2018a), Fuzzy Methods (Musaad O et al., 2020a), AHP + TOPSIS (Chien et al., 2021a), FISM + MICMAC + DEMATEL (Yıldırım et al., 2023), IFMBWM + MULTIMOORA (Shahin et al., 2024), DEMATEL + PROMETHEE II (Torbacki, 2024)	Technological/resource/political barriers persist; government support, regulatory simplification and clean tech are critical for sustainability

#### 3.6.1 Innovation capacity and business strategies

The analysis of the capacity for innovation in companies has been approached from various MCDM methods, highlighting key factors that drive strategic development in different sectors. Matroushi et al. (2018) identify the importance of access to finance and support networks to foster innovation in women-owned businesses in the UAE through the AHP. On the other hand, Rahmanita et al. (2018) and Kiron and Kannan (2018) use FANP and TOPSIS to evaluate adaptive innovation strategies in SMEs and uncertainty in strategic decision-making in technology and manufacturing sectors, respectively, highlighting the need for strategic flexibility. In a similar approach, Grillo et al. (2018) apply cognitive mapping and the Decision Expert (DEX) technique to underscore the role of human capital in innovation, while Gupta and Barua (2018b), using BWM and FTOPSIS, concludes that resource barriers represent a critical obstacle, with government support being a key solution. Chou et al. (2023) and Chen et al. (2022) reinforce the relevance of human capital and financial resources in restaurant and hotel SMEs through FDELPHI, ANP, DEMATEL and DANP (DEMATEL-ANP) models. More recently, Moreira et al. (2024) introduces the concept of frugal innovation and, with AHP, exposes the lack of public policies that promote an innovative culture in regional companies, while Mutlag et al. (2024) identifies with AHP that innovative business environments and organizational capacity are decisive in the growth of SMEs. Together, these studies emphasize the interrelationship between human capital, financing, strategic flexibility, and supportive policies as fundamental pillars for business innovation. In this context, innovation capacity not only emerges as a response to challenges but also plays a transformative role in shaping and enhancing business strategies (Müller et al., 2021). By fostering innovation, companies can align their internal competencies with external opportunities, allowing for more agile, resilient, and competitive strategies (Singh, 2023). The ability to innovate strengthens decision-making under uncertainty, supports differentiation in saturated markets, and enables the efficient allocation of resources–thereby reinforcing strategic planning and execution across diverse organizational settings (Owusu et al., 2024).

#### 3.6.2 Open innovation, evaluation and management

The study of open innovation and its management in SMEs has been approached from various MCDM methods, allowing the identification of key factors for its success. Macedo Filho and Almeida (2018) use AHP and TOPSIS to develop a model for measuring innovation in four dimensions: organization, management, technology, and market. Hakaki et al. (2021) expands this approach with FDEMATEL and ant colony, highlighting the influence of internal and external factors on open innovation in manufacturing SMEs and recommending the strengthening of links with universities and research institutions. Amoozad Mahdiraji et al. (2023) introduces a multi-layered model based on FDELPHI, DEMATEL, ANP, and SWARA to prioritize innovation factors in crowdfunding success, providing an assessment tool to measure an SMEs readiness in this area. In a complementary way, Vakil Alroaia (2023) develops a model for open innovation in business development with ANP and PROMETHEE, identifying that product characteristics play a determining role. Farjam et al. (2023), using BWM and FTODIM, analyzes risk management in open innovation, finding that lack of internal commitment and low adaptability to technological advancement are critical factors in its implementation. Finally, Jing et al. (2024) investigates the selection of lean management innovation strategies in family SMEs with DEMATEL, DANP, and VIKOR, providing a model to evaluate and adopt optimal management schemes. Together, these studies highlight the importance of systematic evaluation and strategic management in open innovation, pointing to the need to strengthen institutional collaboration, mitigate risks, and adopt structured approaches to improve business competitiveness.

In this context, innovation–particularly when developed through open and collaborative processes–emerges as a central mechanism for enhancing competitiveness in SMEs (Carrasco-Carvajal et al., 2023). Open innovation enables firms to transcend internal limitations by incorporating external knowledge, technologies, and perspectives, which accelerates problem solving and shortens innovation cycles (Srisathan et al., 2023). By involving stakeholders such as universities, research centers, customers, or even competitors, SMEs can access a broader spectrum of ideas and resources, leading to more robust product development and better market alignment (Bertello et al., 2022). Moreover, the strategic management of open innovation–through tools like those proposed in the studies above–allows firms to mitigate risks associated with uncertainty and to better allocate their innovation-related investments. Ultimately, the synergy between open innovation and effective management practices increases the firm's ability to respond proactively to market changes, differentiate its value proposition, and sustain a competitive advantage in dynamic environments (Albats et al., 2023; Farjam et al., 2023).

#### 3.6.3 Technological and digital innovation

Technological progress and digital transformation are determining factors for the competitiveness and sustainability of SMEs in an environment of constant change (Zhang et al., 2022). Singh et al. (2019) analyzes the impact of technological innovation using AHP and concludes that technological development is essential for SMEs to face global competition and adapt to market changes. Along the same lines, Enjolras et al. (2020) identifies, through AHP and VIKOR, the key factors that influence technological and organizational innovation, highlighting their impact on business competitiveness. Singh (2023) reinforces this relationship through a fuzzy phase model, demonstrating that technological innovation directly influences product performance and sales performance. On the other hand, Albahri et al. (2023) examines the organizational culture for digital innovation through a decision-making model based on fuzzy images, determining that corporate entrepreneurship is the most influential factor in the adoption of digital innovation in SMEs. These studies together show the need to integrate technological and digital innovation strategies to improve efficiency, competitiveness and adaptability in the current environment.

In this context, technological and digital innovation operate as key enablers of business competitiveness by facilitating process optimization, accelerating product development, and improving customer engagement through data-driven decision-making (Ali, 2024). The adoption of emerging technologies such as artificial intelligence, big data analytics, cloud computing, and the Internet of Things (IoT) allows SMEs to increase operational efficiency, reduce costs, and develop new business models that are more resilient and scalable (Abdul-Yekeen et al., 2024; Ugbebor, 2024; Abrokwah-Larbi and Awuku-Larbi, 2024).

Moreover, digital transformation enhances the agility of SMEs, enabling them to respond rapidly to market shifts and customer preferences. As noted by Fahad and Hamilton (2024), the integration of digital technologies not only improves internal operations but also opens new channels for value creation, thus reinforcing competitive positioning in global markets. This is particularly relevant in post-crisis economies where technological capabilities help SMEs recover faster and adapt more effectively (Abdul-Yekeen et al., 2024). Recent research also underscores the role of digital readiness and innovation capability as mediators between digital transformation and firm performance. According to studies such as those by Valdez-Juárez et al. (2024) and Zhang et al. (2022), firms that invest strategically in digital infrastructure and innovation management experience superior financial performance and long-term sustainability. These findings support the idea that digital innovation is not merely a tool for modernization, but a fundamental strategic asset for competitiveness (Ugbebor, 2024).

#### 3.6.4 Green innovation and sustainability

The adoption of green innovation and sustainability in SMEs faces multiple barriers, but also presents strategic opportunities to improve competitiveness and reduce environmental impact (Chien et al., 2021a). Gupta and Barua (2018a), through BWM and FTOPSIS, identifies that technological and resource barriers are the most significant in the adoption of green innovation, proposing government support as a key solution. In a complementary way, Musaad O et al. (2020a) analyzes sustainable business models with fuzzy methods, highlighting that political barriers play a determining role in optimizing the use of resources. In the energy sector, Chien et al. (2021a) employs a hybrid approach of AHP and TOPSIS to demonstrate that the adoption of clean technologies strengthens business competitiveness. Yıldırım et al. (2023), with FISM, MICMAC and DEMATEL, examines the reasons why many SMEs do not adopt green innovation, providing a conceptual model of the causal relationships between the main barriers.

In other hand Shahin et al. (2024) uses IFMBWM and fuzzy MULTIMOORA to prioritize barriers and solutions, highlighting that simplifying regulatory procedures and promoting shared responsibility in the supply chain are effective strategies to foster sustainability. Finally, Torbacki (2024), through DEMATEL and PROMETHEE II, analyzes sustainability in SMEs and concludes that the implementation of technological innovations in processes and products should be the first action to achieve sustainable innovation. In this context, green innovation not only serves environmental goals, but also enhances long-term business performance by improving operational efficiency, reducing costs, increasing brand value, and opening access to environmentally conscious markets (Thomas et al., 2022). Moreover, when integrated with digital transformation, green innovation fosters eco-efficiency and promotes strategic differentiation, enabling SMEs to respond proactively to environmental regulations and shifting consumer demands (Biondi et al., 2002).

The studies analyzed agree that innovation in SMEs is influenced by the ability to adapt, access to technology and financing. Areas of opportunity are identified in the integration of digital tools, the creation of collaborative innovation ecosystems and the implementation of predictive models to optimize strategies. A approach that combines technological, open, digital, and green innovation–supported by institutional frameworks and strategic management–can significantly increase the competitiveness and resilience of SMEs in a rapidly evolving global environment.

### 3.7 Other issues and approaches evaluated MCDM methods in SMEs

In addition to innovation in general, which is the central theme of this work, the analysis of the literature allowed us to identify other topics closely related to innovation, but which address specific aspects within different business contexts. As shown in Table 4, one of the recurring themes is Industry 4.0 and Digital Transformation, where studies focus on the adoption of Industry 4.0 and the impact of digitalization and Big Data on business processes. Another relevant topic is Sustainability and Green Manufacturing, which includes research on sustainable supplier selection, sustainable manufacturing and the circular economy, as well as energy efficiency and the use of renewable energies as innovative strategies in production.

**Table 4:** Other studies using MCDM methods in SMEs.

**Topic**	**Approach**	**References**
Industry 4.0 and digital transformation	Adoption of Industry 4.0	Chang et al., 2021; Aygün and Satı, 2022; Patel and Vinodh, 2024
	Digital transformation and big data	Maroufkhani et al., 2023; Kumar et al., 2024
Sustainability and green manufacturing	Selection of sustainable suppliers	Tong et al., 2022; Musaad O et al., 2020b
	Sustainable manufacturing and circular economy	Abdullah et al., 2023; Toker and Görener, 2023; Al-Hakimi et al., 2022
	Renewable energy and energy efficiency	Odoi-Yorke et al., 2022; Wu et al., 2019
Risk management and business resilience	Supply chain risks	Mahmud et al., 2021; Babu et al., 2021; Phan et al., 2023
	Crisis management and business resilience	Baydaş, 2022; Satapathy and Mishra, 2022; Kustiyahningsih et al., 2022
Decision making in trade and markets	Market and export strategy selection	Hsu et al., 2020; Jafarian-Moghaddam, 2021; Nguyen, 2022
	Adoption of digital marketing and e-commerce	Ocampo et al., 2017; Akhtar et al., 2022; Yadav et al., 2022
Business management strategies and technology selection	ERP and management tools selection	Rădulescu et al., 2020; Cahyadi, 2019
	Performance evaluation and strategic decision making	Toklu and Taşkın, 2019; Schaefer et al., 2022; Nam et al., 2024

Another relevant aspect identified in the reviewed literature addresses Risk Management and Business Resilience, with studies exploring risks in the supply chain and strategies for crisis management and organizational resilience, demonstrating the key role of innovation in risk mitigation. In the field of decision-making in trade and markets, studies were identified that analyze the selection of market and export strategies, as well as the adoption of digital marketing and e-commerce as key tools for the competitiveness of companies.

Finally, a focus was also observed within the field of Business Management and Technology Selection. Studies were found on selecting enterprise resource planning (ERP) tools and other management systems, as well as performance evaluations and strategic decision-making based on digital tools and innovative models. These findings show that innovation in SMEs is not limited only to the generation of new products or services or that it is only studied in a general way but is also analyzed from specific approaches that seek to improve the efficiency, sustainability, and competitiveness of companies in different sectors based on emerging technologies and MCDM methods.

## 4 Discussion

In recent years, several literature reviews have been published applying MCDM methods to evaluate strategic aspects in organizational settings. For example, Sahoo et al. (2024) identified significant growth in publications on supplier selection, especially since 2010, highlighting the consolidation of MCDM methods in strategic decision-making processes. This growth is particularly concentrated in regions such as Asia and Europe, emphasizing their leadership in the application of these methods. Similarly, our findings indicate that India and China are the countries with the highest volume of publications, reinforcing the Asian leadership already documented by previous studies. Likewise, Chowdhury and Paul (2020) observed a predominance of individual (rather than integrated) MCDM methods in assessments related to corporate sustainability, in contrast to our analysis, where hybridization of several MCDM methods was more frequent.

In the field of project selection, de Souza et al. (2021) also highlight a growing interest in MCDM methods since 2020, with AHP being the most widely used, followed by fuzzy set-based methods. de Almeida et al. (2017), in turn, focused their review on risk management in engineering, also identifying AHP as a central tool. While our SLR likewise found that AHP is the most frequently used MCDM method, none of these studies have specifically addressed the relationship between MCDM methods and the innovation assessment in SMEs, revealing a gap in the literature. In response, our findings provide a new perspective by systematically analyzing the application of MCDM methods in the assessment of innovation in the specific context of SMEs.

Recent literature on innovation in SMEs shows a notable evolution in the use of MCDM methods, from individual applications such as AHP to hybrid approaches that integrate techniques like VIKOR, TOPSIS, DEMATEL, or fuzzy methods. AHP stands out for its ability to decompose complex problems and prioritize criteria, making it a valuable tool for strategic decision-making in highly uncertain environments (Singh et al., 2019). However, studies reviewed–such as those by Matroushi et al. (2018), Singh et al. (2019), Mutlag et al. (2024), and Moreira et al. (2024) agree that its effectiveness can be enhanced when combined with other methods, given its limited sensitivity to ambiguity or interdependence between criteria.

An emerging trend is the development of hybrid models integrating AHP with TOPSIS (Chien et al., 2021a; Macedo Filho and Almeida, 2018), VIKOR (Enjolras et al., 2020), or FAHP and FTOPSIS (Musaad O et al., 2020a), allowing not only for the weighting of criteria but also for a more robust ranking and prioritization of alternatives. These approaches are especially useful in complex contexts such as sustainable innovation or public policy design, where multiple dimensions–economic, social, environmental, and cultural–interact. For instance, Moreira et al. (2024) underscore how the absence of public policies limits frugal innovation, and Gupta and Barua (2018a) highlight the lack of government support as a barrier to green innovation.

The inclusion of frugal innovation (Moreira et al., 2024) in analytical frameworks represents a particularly promising contribution, given its focus on low-cost solutions, adapted to resource-constrained contexts, which are especially relevant for SMEs in developing countries. However, other emerging trends and gaps in the literature are also identified, such as the growing role of digital transformation driving new innovative capabilities through the use of disruptive technologies such as artificial intelligence or the internet of things (Kiron and Kannan, 2018; Macedo Filho and Almeida, 2018; Singh et al., 2019; Singh, 2023; Farjam et al., 2023; Torbacki, 2024). Likewise, sustainability has been consolidated as a transversal axis in the formulation of innovation strategies (Gupta and Barua, 2018a; Musaad O et al., 2020a; Chien et al., 2021b; Yıldırım et al., 2023; Shahin et al., 2024; Torbacki, 2024), although there are still few studies that explicitly integrate environmental variables in MCDM methods applied to SMEs. These gaps open up lines of future research to strengthen the dynamic and contextualized nature of MCDM methods.

On the other hand, the FANP method allows for modeling interdependent relationships between criteria and managing uncertainty, showing strong potential in dynamic and uncertain contexts such as those faced by technology-based SMEs (Kiron and Kannan, 2018). Its integration with TOPSIS, as shown in the study by Rahmanita et al. (2018), demonstrates that combined approaches offer a clearer prioritization structure and improve strategic adaptability, especially in countries with changing institutional contexts such as Indonesia. On the other hand, the DEMATEL method, widely used in studies such as those by Hakaki et al. (2021), Chen et al. (2022), and Amoozad Mahdiraji et al. (2023), stands out for its ability to map causal relationships among factors, facilitating the identification of key drivers of innovation. Its combination with other methods–such as DANP, Fuzzy Delphi, SWARA, PROMETHEE II, or even optimization algorithms like ant colony (Hakaki et al., 2021) enables the construction of multi-layered analytical frameworks that better capture the complexity of innovation systems, including institutional, organizational, and technological factors.

Critically, most studies acknowledge the need to consider both internal and external factors to the organization, where aspects such as organizational culture, strategy, and internal resources intertwine with external variables such as public policies, institutional infrastructure, and economic environment. This comprehensive perspective is especially relevant when analyzing the applicability of MCDM methods in cross-cultural contexts. As suggested by Gupta and Barua (2018a), public policies and institutional factors significantly condition innovation–an observation confirmed by our findings, as many studies overlook this component. Countries such as Brazil, Saudi Arabia, or Turkey present very different institutional contexts that must be considered in MCDM methods to ensure their applicability.

For future research, it is recommended to move toward adaptive models that integrate dynamic data and machine learning, as well as to explore participatory methodologies that incorporate the perspectives of multiple stakeholders (entrepreneurs, policymakers, academic institutions). It is also suggested to deepen the evaluation of public policies as explicit variables in MCDM methods, in order to improve the formulation of innovation strategies tailored to each socioeconomic context.

It is worth noting that the studies reviewed show that the value of MCDM methods lies not only in their analytical rigor but also in their capacity for adaptation, integration, and contextualization. Their combination with fuzzy approaches, causal networks, or optimization algorithms represents a necessary methodological evolution to address the complex and multidimensional challenges of innovation in SMEs. The inclusion of cross-cultural perspectives and critical analysis of public policies emerge as key avenues to strengthen the applicability and effectiveness of these methods in designing sustainable, inclusive, and strategically relevant solutions.

## 5 Limitations

Although this systematic literature review provides meaningful insights into the application of MCDM methods for innovation assessment in SMEs, several limitations must be acknowledged.

First, the use of the PRISMA methodology, while rigorous and structured, posed certain challenges. The diversity of terminology used across studies–especially in relation to innovation, decision-making, and SMEs–may have led to the exclusion of relevant articles due to inconsistent keyword usage or differences in how studies are indexed across databases. Despite employing Boolean operators, wildcard symbols, and multiple combinations of search terms, semantic variability remains a constraint in ensuring complete coverage.

Second, the review was conducted using publications indexed in selected academic databases and restricted to studies published between 2018 and 2024. This temporal scope, while chosen to reflect recent advances in both innovation frameworks and MCDM methods, may have excluded earlier foundational studies that could provide historical context or methodological baselines.

Third, only studies published in English were included, which may have resulted in language bias, particularly relevant considering the high number of SME-related studies emerging from non-English-speaking countries. Consequently, potentially valuable regional insights may have been omitted, particularly in Latin America, Eastern Europe, or Africa.

Fourth, this review focused primarily on peer-reviewed journal articles and conference proceedings, excluding gray literature, such as technical reports, policy papers, or doctoral dissertations. These sources, while often lacking peer review, may contain applied insights or novel methodologies relevant to practitioners and policymakers.

Fifth, the analysis was primarily descriptive and qualitative, focusing on identifying trends, gaps, and thematic patterns. While this approach is well suited for exploratory purposes, quantitative bibliometric techniques (e.g., co-citation analysis, keyword co-occurrence) were only referenced and not fully implemented in this stage. A future bibliometric extension could provide a more nuanced understanding of influential authors, networks, and research clusters.

Finally, while the review identifies the prevalence and complexity of various MCDM methods, no empirical testing or performance benchmarking of these methods was conducted in real-world SMEs contexts. As such, the practical implications of the comparative strengths and limitations of MCDM methods remain theoretical and warrant further validation through case studies or simulations.

Recognizing these limitations provides a clearer pathway for future research and underscores the importance of methodological pluralism and contextual sensitivity when conducting systematic reviews in emerging, multidisciplinary fields.

## 6 Conclusions

This systematic literature review has explored the application of MCDM methods in assessing innovation and other strategic factors in SMEs during the 2018–2024 period. The research process followed a sequential and structured path that enhances transparency and reproducibility while allowing for a clear articulation of the study's contributions.

The review began with the formulation of two guiding research questions: What are the main trends and findings in the application of MCDM methods for innovation assessment in SMEs? And, what other factors besides innovation have been evaluated in SMEs using MCDM methods? These questions emerged from the increasing complexity SMEs face in responding to economic, technological, environmental, and social pressures (Cosenz and Bivona, 2021), and from the growing interest in structured approaches to support decision-making under such conditions (Taherdoost and Madanchian, 2023).

The document search and selection process followed a rigorous protocol based on the PRISMA method and the systematic review guidelines proposed by Kitchenham et al. (2010), which are widely adopted in technical and applied research. An initial identification of 8,543 records was obtained from various scientific databases. However, due to access limitations, only 694 full-text documents were retrieved. These were subjected to predefined exclusion criteria related to duplication, thematic relevance, methodological quality, and availability of essential information. As a result, 25 peer-reviewed articles that fully met the inclusion criteria were selected for in-depth analysis. Tools such as Publish or Perish and VOSviewer were also employed to support bibliometric mapping and facilitate the identification of relevant research clusters within the selected corpus.

Subsequently, a descriptive and thematic analysis was conducted to examine trends by publication year, geographic origin, and sectoral focus. The results revealed a concentration of studies in India and China, reflecting an emphasis on emerging economies where innovation in SMEs is frequently shaped by state-led development agendas and rapid economic transformation. The most commonly studied sectors were the business and academic spheres, followed by the social sector. In terms of innovation, four principal categories emerged: innovation capacity and business strategies; open innovation, evaluation, and management; technological and digital innovation; and green innovation and sustainability. Additionally, the review identified five broad themes and eleven specific factors assessed with MCDM methods beyond innovation, including financial performance, supply chain efficiency, sustainability metrics, risk management, and customer satisfaction.

From a theoretical standpoint, this review contributes by consolidating fragmented knowledge and synthesizing methodological patterns across contexts. It highlights a strong preference for fuzzy MCDM methods and hybrid models, demonstrating the importance of managing uncertainty and multidimensionality in SMEs decision-making environments. Furthermore, the increasing integration of MCDM methods with emerging technologies–such as artificial intelligence, simulation tools, and data analytics–suggests a methodological evolution toward more automated and scalable decision support systems.

On the managerial level, the findings offer concrete insights for SMEs leaders seeking to enhance their innovation strategies and resilience. MCDM methods facilitate more informed and participatory decision-making, support strategic resource allocation, and improve firms' ability to adapt to changing market conditions (Taherdoost and Madanchian, 2023). The evidence also underscores the value of co-created knowledge and collaborative evaluation frameworks in strengthening SMEs' adaptive capacity.

For public policy, the review underscores the potential benefits of incorporating MCDM methods into SMEs development programs, particularly in developing economies where uncertainty and resource constraints are more acute (Muriithi, 2017). Policymakers could adopt MCDM-based approaches for innovation grant selection, program evaluation, and ecosystem planning, thereby promoting more rational, transparent, and context-sensitive decision-making.

It is important to reflect on the geographic and sectoral distribution of the reviewed studies. The dominance of research from Asia points to a potential regional bias, and the limited presence of studies from Latin America and Africa signals the need for broader geographic inclusion. Similarly, the concentration of case studies in business and academic environments suggests an opportunity to expand MCDM methods applied to sectors such as agriculture, services, and informal economies, which are often central to the development trajectories of low- and middle-income countries.

Looking ahead, future research should aim to adapt MCDM methods more closely to the realities of SMEs, validate hybrid models in real business scenarios, and explore sector-specific applications in underrepresented regions. There is also a promising frontier in aligning MCDM methods approaches with digital transformation strategies to build more agile, data-informed (Kumar et al., 2024). In sum, the effective use of MCDM methods in SMEs contexts requires a multidisciplinary, flexible, and collaborative approach. This review provides evidence that these methods can significantly enhance innovation capacity, resilience, and long-term sustainability for SMEs operating in diverse and dynamic environments.

## Data Availability

The datasets presented in this study can be found in online repositories. The names of the repository/repositories and accession number(s) can be found in the article/supplementary material.
